# Assessment of Serum Omentin-1 and Interleukin-6 in the Diagnosis of Early Stages of Diabetic Nephropathy: A Cross-Sectional Observational Study

**DOI:** 10.7759/cureus.64239

**Published:** 2024-07-10

**Authors:** Sujata Devi, Suchanda Sahu, Kishore Behera, Nibedita Priyadarshini, Debananda Sahoo

**Affiliations:** 1 Internal Medicine, All India Institute of Medical Sciences, Bhubaneswar, Bhubaneswar, IND; 2 Biochemistry, All India Institute of Medical Sciences, Bhubaneswar, Bhubaneswar, IND; 3 Endocrinology, All India Institute of Medical Sciences, Bhubaneswar, Bhubaneswar, IND; 4 Physiology, All India Institute of Medical Sciences, Bhubaneswar, Bhubaneswar, IND

**Keywords:** omentin 1, interleukin 6, albuminuria, cytokines, diabetic nephropathies

## Abstract

Introduction

The pathogenesis of diabetic nephropathy highlights the progression of inflammation and fibrosis from tubular to glomerular damage during the early stages of kidney involvement in diabetic individuals. As urine albumin serves as a marker for glomerular function, its detection indicates a stage of diabetic nephropathy where the glomerulus is already compromised. Consequently, relying solely on urine albumin for diagnosis becomes questionable. In our pursuit of identifying innovative biomarkers for the early detection of diabetic nephropathy, this study was crafted to explore the relationship between chemokines, omentin-1, interleukin-6, and microalbuminuria.

Materials and methods

Our study cohort comprised 116 patients diagnosed with diabetes mellitus. In our study, participants were stratified into two groups based on their urine albumin levels: Group 1, characterized by urine albumin creatinine ratio <30 mg/gm and estimated glomerular filtration rate >90 ml/min, and Group 2, with urine albumin creatinine ratio ≥30 mg/gm and <300 mg/gm, and estimated glomerular filtration rate >60 ml/min and <90 ml/min. Serum creatinine, glycated hemoglobin (HbA1c), fasting blood sugar and post-prandial blood sugar, lipid profile, total protein, albumin, fasting insulin, omentin-1, and interleukin-6 were estimated.

Result

There was a significant difference in the medians of serum urea, creatinine, omentin-1, interleukin-6, urine albumin creatinine ratio, and estimated glomerular filtration rate levels in the two groups. There was no difference in fasting blood sugar, post-prandial blood sugar, HbA1c, serum lipids, fasting insulin, and homeostatic model assessment for insulin resistance. The receiver operating characteristic curve plotted for the newer biomarkers of diabetic nephropathy showed that there was a significant diagnostic utility in diabetic nephropathy detection of serum omentin (p=0.000), interleukin-6 (p=0.002), and interleukin-6: omentin-1 ratio (p=0.000), which correlated well with the routine test that is urine microalbumin estimation. Risk assessment demonstrated that type 2 diabetes mellitus patients with an interleukin-6: omentin-1 ratio ≥0.26 had significantly higher odds, with an odds ratio of 3.97, for developing diabetic nephropathy, which was statistically significant. Conversely, a ratio of ≤0.26 was associated with kidney protection among patients with type 2 diabetes mellitus.

Conclusion

Our findings revealed decreased levels of omentin-1 and increased levels of interleukin-6 in the group with diabetic nephropathy compared to those without diabetic nephropathy among patients with type 2 diabetes mellitus. Interleukin-6: omentin-1 ratio of ≤0.26 was associated with kidney protection among patients with type 2 diabetes mellitus. Based on the results obtained from this study, we propose that measuring the serum interleukin-6: omentin-1 ratio in patients with type 2 diabetes mellitus may assist in identifying the early stages of diabetic nephropathy before the onset of microalbuminuria. Timely intervention in these patients predisposed to diabetic nephropathy can aid in better treatment outcomes in type 2 diabetes mellitus.

## Introduction

According to the International Diabetic Federation (IDF), there are 463 million diabetic adults globally, of which 77 million alone are in India in 2020 [1]. Thirty-six percent of people with diabetes develop diabetic nephropathy (DN), and it is usually detected in stage 3 chronic kidney disease (CKD) or as an end-stage renal disease (ERDS) [1,2]. In India, DN is the most common cause of CKD [3]. DN in people with diabetes is detected early by urine microalbumin, which is characterized by quantitative estimation of urine albumin of 30-300 mg/ day or as urine albumin creatinine ratio (UACR) between 30 and 300 mg/gm. Early detection of microalbuminuria and prompt intervention can halt or even reverse the advancement of nephropathy [4]. Studies have shown that urine albumin in people with diabetes is altered due to the glycation and attachment of fatty acids and its partial hydrolysis in the tubular lumen [5-7]. These alterations make the urine albumin non-reactive with the immune assay methods used conventionally for its detection [8].

Recent understandings of the pathogenesis of DN indicate that inflammation and fibrosis progress from tubular to glomerular damage during the initial stages of kidney involvement in diabetics [9]. Since urine albumin serves as a marker for glomerular function, its detection signifies a stage of DN where the glomerulus is already compromised. Therefore, the reliability of urine albumin estimation becomes questionable.

In our pursuit of identifying newer and more effective biomarkers for the early detection of DN, this study was crafted to explore the correlation between chemokines, omentin-1, and interleukin-6 (IL-6) with microalbuminuria. Omentin-1 is an adipokine synthesized by the endothelial cells of visceral adipose tissue. It has anti-inflammatory activity associated with micro and macrovascular complications of diabetes mellitus [10]. Interleukin-6 is increased in an inflammatory response in the progression of DN [11]. Therefore, the objective of the study was to estimate serum omentin-1 and IL-6 levels in type 2 diabetes mellitus (T2DM) with early stages of nephropathy.

This article was previously presented as a meeting abstract at the 77th Annual Conference of the Association of Physicians of India (APICON 2022) conducted on 14th-17th April 2022.

## Materials and methods

Study setting and sample size

This cross-sectional observational study was a collaborative effort between the Departments of General Medicine and Biochemistry at the All India Institute of Medical Sciences, Bhubaneswar, India, conducted from March 2019 to February 2020.

The sample size required for the study was estimated as 116 type 2 diabetes mellitus (T2DM) patients, 58 patients without microalbuminuria in group 1 and 58 patients with microalbuminuria in group 2. The same was calculated to detect the difference of 5 ng/ml in omentin-1 with a standard deviation of 9.5 at a 5% level of significance and 80% power [12].

Method

A total of 180 T2DM patients were recruited from the General Medicine Outpatients Department of the All India Institute of Medical Sciences, Bhubaneswar. After clinical examination and initial laboratory investigations, 64 were excluded due to comorbidities. They were classified into two groups according to their urine microalbuminuria, which was measured by the urine albumin to creatinine ratio in the spot urine sample. Group 1 were with negative microalbuminuria (<30 mg/gm) and estimated glomerular filtration rate (eGFR) >90 ml/min; Group 2 were with positive microalbuminuria (30-300 mg/gm) and eGFR >60 ml/min but <90 ml/min. Patients with UACR >300 mg/gm, pregnancy, comorbidities like thyroid disorders, disorders requiring the long-term use of anti-inflammatory drugs or steroids, and type 1 diabetes mellitus were excluded from the study. Patients with proteinuria due to any other cause were also excluded from the study. 

Clinical data were gathered after obtaining written consent. Serum creatinine, glycated hemoglobin (HbA1c), fasting blood sugar (FBS) and post-prandial blood sugar (PPBS), lipid profile, total protein, albumin, and fasting insulin were estimated on the same day. All colorimetric estimations were conducted on the same day utilizing the Beckman Coulter Chemistry Analyzer AU5800 (Beckman Coulter, Brea, USA). Fasting insulin levels were determined by chemiluminescence immunoassay (CLIA) using ADVIA Centaur XP, Siemens Healthcare Diagnostics Inc, Germany. The serum was separated and stored in two separate 1 ml aliquots at −20°C until assay. The following assays were performed on stored serum samples: Serum Omentin-1 (Sincere Biotech, Beijing, China) and IL6 (Fine Test, Wuhan Fine Biotech Co. Ltd., Wuhan, China) were measured using the enzyme-linked immunosorbent assay (ELISA) method following the manufacturer's instructions. Urine samples were collected as mid-morning void midstream portions. Urine albumin was determined by the immune-turbidimetric method, and urine creatinine by Jaffe's kinetic method using an auto-analyzer from Beckman Coulter Chemistry Analyzer AU5800 (Beckman Coulter, Brea, USA). The calculated parameters were urine albumin creatinine ratio, homeostatic model assessment for insulin resistance (HOMA-IR) [13], and estimated glomerular filtration rate (eGFR) [14]. 

Statistical analysis

The data was not normally distributed (Kolmogorov-Smirnov test applied), it was represented as median and interquartile ranges (IQR). The outcome variables of the two groups were analyzed by the Mann-Whitney U test. A receiver operating characteristic (ROC) curve was used to analyze omentin-1, IL-6, IL-6: omentin-1 with HOMA-IR and UACR. Fisher’s Exact Test was applied to compare the proportion of IL-6: omentin-1 between the two groups. Statistical analysis was done using the SPSS 19.0 version (IBM Corp., Armonk, New York, USA) for Windows.

Ethical statement

Ethical approval was obtained from the Institute Ethics Committee (T/IM-F/18-19/27). Informed consent was obtained from all the study participants.

## Results

Our study cohort consisted of 116 patients diagnosed with diabetes mellitus. They were categorized into two groups based on their urine albumin levels: group 1 comprised individuals with UACR <30 mg/gm and eGFR >90 ml/min, while group 2 included those with UACR ≥30 mg/gm and <300 mg/gm and eGFR >60 ml/min and <90 ml/min. 

Table 1 shows the general characteristics and the biochemical parameters compared in the two groups. There was a significant difference in the medians of serum urea, creatinine, omentin-1, IL-6, UACR, and eGFR levels in the two groups. There was no difference in age, FBS, PPBS, HbA1c, serum lipids, fasting insulin, and HOMA-IR.

**Table 1:** Comparison of general characteristics and biochemical parameters between the two study groups. IQR: interquartile range, SBP: systolic blood pressure, DBP: diastolic blood pressure, FBS: fasting blood sugar, PPBS: post-prandial blood sugar, urine ACR: urine albumin creatine ratio, eGFR: estimated glomerular filtration rate, IL6: interleukin-6, S. alb: serum albumin, T chol: total cholesterol, TG: triglyceride, HDL: high-density lipoprotein, VLDL: very-low-density lipoprotein, LDL: low-density lipoprotein, HOMA-IR: homeostatic model assessment for insulin resistance.

Groups	Group 1 (controls) n=58			Group 2 (cases) n=58			P-value
Variables	Median	IQR		Median	IQR		
		25th	75th		25th	75th	
Age (years)	54.5	46.8	62.3	54.5	45.0	63.0	0.958
SBP (mm of Hg)	133.5	120.0	142.0	130.0	120.0	145.5	0.932
DBP (mm of Hg)	80.0	75.3	90.0	80.0	70.0	86.0	0.316
Pulse (/min)	80.0	77.5	86.5	80.0	80.0	88.0	0.136
FBS (mg/dl)	124.0	108.8	154.3	137.3	110.8	160.0	0.288
PPBS (mg/dl)	199.0	162.8	254.3	197.5	165.8	247.0	0.976
HbA1C (%)	7.5	6.8	8.3	7.8	6.8	9.0	0.390
S. urea (mg/dl)	20.0	18.0	23.0	23.0	20.0	24.3	0.006
S. creatinine (mg/dl)	0.7	0.7	0.8	1.0	0.9	1.1	0.000
Urine ACR (mg/gm)	16.7	12.1	24.4	70.9	46.5	91.8	0.000
eGFR (ml/min)	99.9	96.0	109.3	71.9	63.9	78.3	0.000
Omentin-1 (pg/ml)	306.1	189.5	557.5	67.2	31.7	196.2	0.000
IL-6 (pg/ml)	29.7	17.1	57.7	37.9	29.5	80.8	0.002
F-insulin (μg/ml)	8.6	4.6	16.6	11.6	5.3	25.1	0.124
S. protein (g/dl)	7.6	7.4	7.8	7.5	7.3	7.7	0.263
S. Alb (g/dl)	4.4	4.2	4.6	4.4	4.2	4.6	0.742
T chol (mg/dl)	177.0	149.5	205.3	189.0	164.0	210.3	0.120
TG (mg/dl)	155.5	126.8	193.5	166.5	131.8	216.0	0.156
HDL (mg/dl)	45.0	38.0	50.0	45.0	39.0	50.3	0.730
VLDL (mg/dl)	28.0	22.8	36.0	27.5	21.5	35.0	0.879
LDL (mg/dl)	107.0	81.0	121.8	114.0	89.8	134.0	0.078
HOMA-IR	2.9	1.5	5.7	3.8	1.9	9.5	0.229

**Figure 1:**
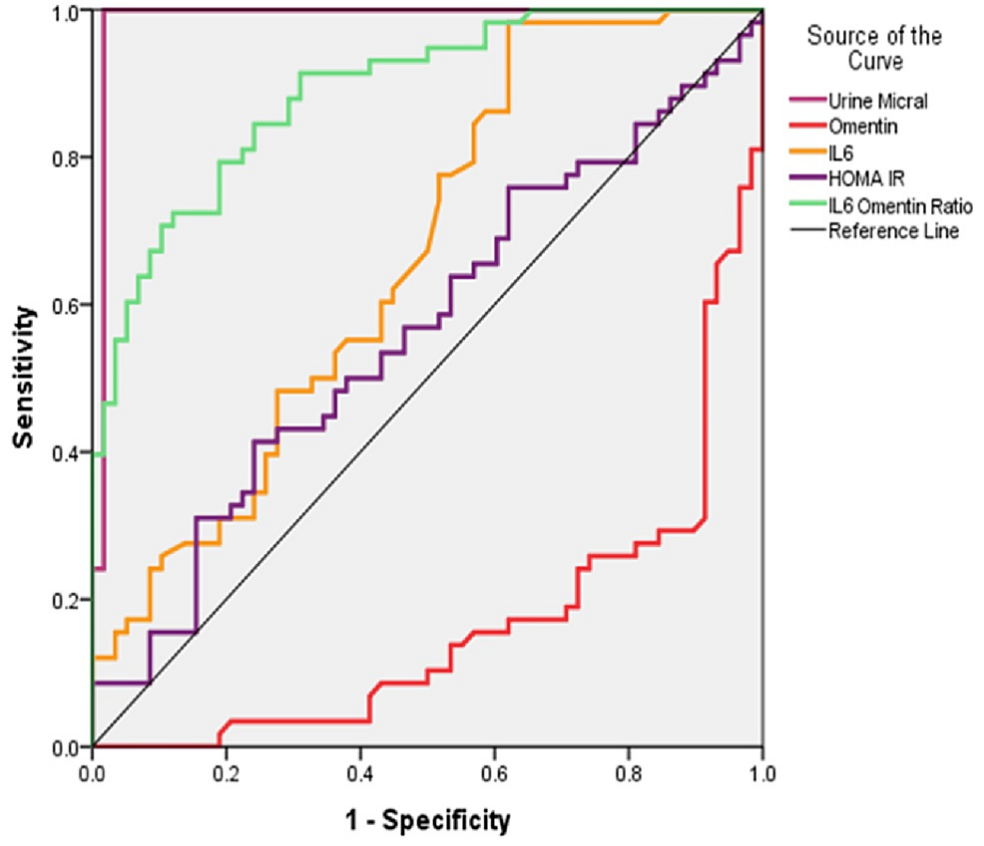
ROC curve for the different biochemical parameters in diabetic nephropathy. ROC: receiver operating characteristics, urine micral: urine microalbuminuria test, IL6: interleukin-6, HOMA-IR: homeostatic model assessment for insulin resistance.

The ROC curve plotted for the newer biomarkers of diabetic nephropathy (Figure 1) and Table 2 showed that there was a significant diagnostic utility in DN detection of serum omentin (p=0.000), IL-6 (p=0.002), and IL-6: omentin-1 ratio (p=0.000), which correlated well with the routine test that is urine microalbumin estimation. From the ROC curve, we obtained cut-off values for the serum biomarkers. Table 3 shows the cut-off levels of the newer biomarkers of DN. IL-6: omentin-1 ratio has better sensitivity and specificity than other parameters. Considering our study groups, we segregated subjects based on the IL-6: omentin-1 ratio.

**Table 2:** Area under the curve obtained from ROC for different biochemical parameters. ROC: receiver operating characteristics, urine ACR: urine albumin creatinine ratio, IL6: interleukin-6, HOMA-IR: homeostatic model assessment for insulin resistance.

Area under the curve					
Test result variable (s)	Area	Std. error	Asymptotic sig.	Asymptotic 95% confidence interval	
				Lower bound	Upper bound
Urine ACR (mg/gm)	0.987	0.013	0	0.961	1
Omentin-1 (pg/ml)	0.162	0.037	0	0.089	0.235
IL-6 (pg/ml)	0.666	0.05	0.002	0.567	0.764
HOMA IR	0.565	0.054	0.229	0.46	0.67
IL-6: omentin-1 ratio	0.889	0.029	0	0.832	0.946

**Table 3:** Cut-off levels of biomarkers for diabetic nephropathy obtained from ROC. ROC: receiver operating characteristics, IL6: interleukin-6, HOMA-IR: homeostatic model assessment for insulin resistance.

	Cut-off from ROC	Sensitivity (%)	Specificity (%)
Omentin-1 (pg/ml)	175.9	74	81
IL-6 (pg/ml)	30.75	60	57
HOMA-IR	3.07	57	53
IL-6: omentin-1 ratio	0.26	79	81

Table 4 shows the comparison of proportions of the two groups, the IL-6: omentin-1 ratio >0.26 was present in 81% of the group 2 subjects, that is, in T2DM with microalbuminuria (the DN group), which was statistically significant (p=0.000). The risk estimates calculated (Table 5) demonstrated that diabetes mellitus patients with an IL-6: omentin-1 ratio ≥0.26 had significantly higher odds, with an odds ratio of 3.97, for developing DN. Conversely, a ratio of ≤0.26 was associated with kidney protection among patients with diabetes mellitus.

**Table 4:** The proportion of diabetic nephropathy subjects using the IL-6: omentin-1 ratio cut-off. IL-6: interleukin-6.

IL-6: omentin-1 ratio	Group		Total	Fisher's Exact test, P-value
	1 (Control)	2 (Cases)		
<0.26	46 (79.3%)	11 (19%)	57	0.000
>0.26	12 (20.7%)	47 (81%)	59	
Total	58	58	116	

**Table 5:** The risk estimates of the IL-6: omentin-1 ratio in the development of diabetic nephropathy. IL-6: interleukin-6.

Risk estimate			
	Value	95% Confidence interval	
		Lower	Upper
Odds ratio for IL-6: omentin-1 ratio category (groups 1 and 2)	0.061	0.024	0.152
For cohort group=1	0.242	0.14	0.418
For cohort group=2	3.968	2.357	6.679
Number of valid cases	116		

## Discussion

In this study, we had planned to study the association of omentin-1 and IL-6 with microalbuminuria, the hallmark of DN, and assess the utilization of these newer serum biomarkers in the detection of DN. The main findings of our study were as follows: First, there was no difference in the age, FBS, PPBS, HbA1c, fasting insulin, HOMA-IR, and lipid profile in the two groups. A large cohort study showed similar results-Veterans Affairs Trial-Follow-up study (VADT-F) in which though there was no difference in the HbA1c, lipid profile, and blood pressure in the two groups, the kidney disease outcome was better with more intensive glycaemic control treatment than in those with standard treatment [15]. Secondly, the renal function tests, which included serum urea, creatinine, and eGFR, were significantly different in the two groups, along with the new biomarkers omentin-1 and IL-6. The ROC curve showed the IL6: omentin-1 ratio as a good marker for DN, followed by serum omentin-1 and IL-6. Lastly, the IL-6: omentin-1 ratio was able to differentiate DM patients predisposed to DN, considering an eGFR as 90 ml/min as the cut-off for diminished kidney function.

Our study findings indicated that levels of omentin-1 were diminished, while IL-6 levels were elevated in the group with DN compared to those with type 2 diabetes mellitus without DN. Our observation was similar to the studies done by Tekce et al. [16] and Senthilkumar et al. [12]. The increased risk of T2DM in Asian Indians has been attributed to visceral adiposity [17] and the subsequent risk involved in atherogenicity, renal, and cardiovascular involvement [18]. Omentin-1, a chemokine from the visceral adipose tissue, acts by increasing glucose uptake and maintaining insulin sensitivity in the physiological state. Whereas in T2DM, which is due to insulin resistance, the omentin-1 levels are seen to decrease. Its lowered circulating levels cannot function as an optimal anti-inflammatory mediator, tipping the effect towards inflammation [19]. The concurrent rise in IL-6 by the endothelial cells is in response to hyperglycemia, resulting in chronic inflammation [20]. Therapeutic strategies should target the restoration of omentin-1 through diet, exercise, and drugs [19]. The negative correlation of circulating omentin-1 with IL-6 is probably related to the pathogenesis of DN [21]. As seen in our study, these serum biomarkers are comparable with UACR. The IL-6: omentin-1 ratio was able to identify that subset of diabetics with a decrease in kidney function irrespective of the UACR status. This type of study has not been reported so far. Choudhary et al. observed that the inflammatory markers IL-6 and C-reactive protein (CRP) are responsible for inflammatory damage to kidneys in DN, irrespective of the presence of urine albumin [22]. A meta-analysis study by Chen et al. has suggested the specific polymorphic forms of IL-6 associated with DN [23]. IL-6 progressively increased in serum and urine in T2DM from normal to micro- to macroalbuminuria [24].

The strengths of our study are that we have thoroughly examined our subjects for other comorbidities, thereby avoiding confounding factors that may affect renal function, and that our study participants were similar in general characters in the two groups compared. The limitations were that our study included consecutive convenient sampling that was hospital-based. Larger, multi-centric cohort studies are suggested for the establishment of cut-off levels for these newer biomarkers to identify that subsection of T2DM patients who are at risk for DN before the onset of microalbuminuria.

## Conclusions

Our study findings indicated that levels of omentin-1 were diminished, while IL-6 levels were elevated in the group with DN compared to those with type 2 diabetes mellitus without DN. The ROC curve showed IL-6: omentin-1 ratio as a good marker for DN, followed by serum omentin-1 and IL-6. The IL-6: omentin-1 ratio was able to differentiate DM patients predisposed to DN, considering an eGFR as 90 ml/min as the cut-off for diminished kidney function. Based on the results obtained from this study, we propose that measuring the serum IL-6: omentin-1 ratio in patients with type 2 diabetes mellitus may assist in identifying the early stages of diabetic nephropathy before the onset of microalbuminuria. Timely intervention in these patients predisposed to diabetic nephropathy can aid in better treatment outcomes in type 2 diabetes mellitus.
